# Accessing mental health walk-in clinics and other services for children and families

**DOI:** 10.1177/27550834231186682

**Published:** 2023-07-31

**Authors:** Catalina Sarmiento, Graham J Reid

**Affiliations:** 1 Department of Psychology, The University of Western Ontario, London, ON, Canada; 2 Departments of Psychology, Family Medicine, and Paediatrics, The University of Western Ontario, London, ON, Canada; 3 Children’s Health Research Institute, London, ON, Canada

**Keywords:** Mental health walk-in clinics, children, youth, Ontario, Canada

## Abstract

**Background:**

Mental health walk-in clinics (MHWCs) are a model of service delivery that has gained increasing interest and traction. The aim of the study was to better understand how MHWC use is related to use of other services provided by agencies.

**Objectives:**

(1) Explore if and how MHWCs are used alongside other services, including the different time points (e.g. MHWCs used exclusively, MHWCs used before other agency services); (2) identify correlates of MHWC use alongside other agency services.

**Design:**

Administrative data from two child and youth mental health agencies in Ontario were extracted, including demographics, visit data, and presenting concerns.

**Methods:**

In this exploratory, descriptive study, analyses of administrative data were conducted to identify patterns and correlates of MHWC use before other agency services, compared with MHWC use exclusively.

**Results:**

More than half of families used MHWCs and other agency services before or concurrently with other agency services. Child age, guardianship, and disposition at discharge emerged as correlates of MHWC use before other agency services.

**Conclusions:**

MHWCs are sufficient for some families, easing the pressure on other agency services. For the remaining families, MHWCs can help support them at the beginning of their service use journey.

## Introduction

Access to mental health services remains difficult for children and families with the rising demand for services^1^ and lengthy waitlists.^2^ Single session therapy (SST) is a promising service delivery model to help address this issue.^3^ SST treats each session as if it will be the only session with the client.^4^ There are two ways in which SST has been used: scheduled and unscheduled (i.e. MHWCs). MHWCs do not require phone calls to schedule appointments, attending comprehensive intakes, or waiting days to weeks to start services.^3,5^ A provincial survey conducted in 2020 examined the implementation of MHWCs in child and youth mental health (CYMH) agencies in Ontario.^6^ This survey found that this service delivery model is flexible, using different approaches (e.g. narrative therapy, solution-focused, therapy, and cognitive behavioral therapy) and modalities (e.g. consulting break and outsider witness). Most agencies have social workers or registered psychotherapists who provide MHWCs from more than one location (e.g. agency, school community center, and doctor’s office). Multiple locations likely make it easier for families to access them.

Scheduled SST has been studied extensively, which has allowed for literature reviews and meta-analyses to be conducted.^4,7^ Previous research supports its use for internalizing concerns (e.g. anxiety), externalizing concerns (e.g. conduct problems and substance use), and other concerns (e.g. general distress);^4,7^ MHWCs have been studied to a lesser extent. Resent research has found perceived improvement immediately after the MHWC session (e.g. less distress, greater confidence, and more hopefulness)^8,9^ and improvement at follow-up (e.g. lower psychopathology and lower distress).^8,10,11^ Both scheduled SST and MHWCs have been found to have high client satisfaction.^5,8,12–14^

MHWCs are part of a continuum of services provided by CYMH agencies.^8^ The role that this service delivery model plays within the continuum is largely unknown. This study examines this gap in the literature.

The following sections briefly review the theoretical framework and relevant service use research.

### Theoretical framework

The Revised Network-Episode Model^15^ was developed to conceptualize mental health service use by children (0–18 years) and their families. The model outlines four broad factors, with 76 variables nested within the factors, that influence mental health service use: (1) social content (e.g. child age, child gender, parental income, and parental marital status), (2) social support system, (3) illness career (e.g. termination of care and referrals), and (4) treatment system. This model was used to frame this study. More specifically, to guide the selection of correlates and how they would be entered into the regression models.

### MHWC service use

There is limited research examining how MHWCs relates to other services families may access. Some studies have found that following a MHWC visit, families have less service use at follow-up across mental health, education, juvenile justice, and general medical sectors;^10,11^ however, this is not a consistent finding. Horton et al.,^16^ for example, found that clients that attended a MHWC used more services following their MHWC visit compared to before their MHWC visit. The authors hypothesized that MHWCs may serve as a source for referrals and system navigation. This is supported by other research that shows that between 8% and 25% of MHWC clients are referred to other services.^11,14^ All previous studies used select samples and relied on patient/parent reports, and many reported substantive difficulties with participant recruitment and retention.^10,16^

### Study

The aim of the study was to understand how MHWC use is related to the use of other services provided by agencies. We used administrative data to overcome limitations from previous studies that used selected samples and parent/patient self-report. There were two objectives: (1) explore if and how MHWCs are used alongside other services, including the different time points (e.g. MHWCs used exclusively, MHWCs used before other agency services); (2) identify correlates of MHWC use alongside other agency services. The second objective was clarified and informed by the results of the first objective, which allowed for a better understanding of this phenomenon.

It was hypothesized that some families use only MHWCs and some families use MHWCs alongside other agency services. For the latter, MHWCs are likely to be the first service received from an agency and can support families as they are connected to other agency services.

## Methods

The study was conducted in Ontario, Canada. This is the first study to examine how visits to MHWCs are related to use of other mental health service(s), using administrative data from CYMH agencies. As such, an exploratory, descriptive methodology was used.^17,18^ In the absence of similar studies in the literature, it was not possible to conduct a-priori power/sample size calculations.

### Recruitment and sampling strategy

#### Inclusion and exclusion criteria for agencies

The mental health service network for children and families in Ontario is complex.^19^ Children and families can receive services from different sectors, including health (e.g. CYMH agencies, family health teams, hospitals, pediatricians, and psychiatrists), education, child welfare, juvenile justice, and private (e.g. psychologists and social workers) sectors.^20^ The pathway to access these services varies (e.g. self-referral for CYMH agencies; family physician referral for pediatricians and psychiatrists) as does the funding/cost (e.g. publicly funded CYMH agencies; fee-for-service psychologists and social workers).^20^ It is also important to note that there are significant differences in the service mandates even within the same sector and type of mental health provider.^20^ For example, CYMH agencies in the health sector can serve the entire community (e.g. population living within a delimited geographic area) or a subset of the community (e.g. subset of a population, like Indigenous, living within a given geographic area), and children of all ages (i.e. birth–18 years) or a subset of children (e.g. birth–12 years).^17^ An agency can provide services for most mental health concerns (e.g. anxiety, depression, attention, hyperactivity, and noncompliance) or only for specific concerns (e.g. addictions only). Services may also be offered in a variety of different formats/settings (e.g. phone *and* face-to-face; outpatient *and* residential) or only in one format/setting (e.g. phone only and residential care only).^19^

This study sought to identify CYMH agencies: serving all children (i.e. birth to 18 years) in the entire community (i.e. population living within a given geographic area), providing services for most mental health concerns (e.g. anxiety, depression, attention, hyperactivity, and noncompliance), and delivering services in a range of formats and settings (e.g. phone *and* face-to-face; outpatient *and* residential), and located in a census division with a small urban center. This size for urban centers^21^ was selected for two main reasons. First, the population is large enough to yield sufficient data. Second, previous work has demonstrated that large urban areas in Ontario, metropolitan Toronto/Greater Toronto Area, in particular, have more CYMH agencies in a given area and higher prevalence of children and youth using services.^22^ As such, families in these areas may receive services from multiple agencies, which would be difficult to account for. This would bias the results as some families would experience the event of interest (i.e. MHWC use and other agency use), but they would be misclassified (i.e. MHWC use only).

There were five inclusion criteria for the agencies: (1) serve children birth–18 years, (2) no fees for mental health services, (3) providing face-to-face services (pre-COVID-19 pandemic), (4) providing MHWCs before 2020 (i.e. pre-COVID-19 pandemic), and (5) located in a census division with a small urban center (population between 50,000 and 200,000), given higher access within large urban areas noted above.

There were five exclusion criteria for the agencies: (1) primarily focus on specific disorders (e.g. addictions, developmental disorders, disabilities, bereavement, palliative care, health, and criminality/justice system), (2) provides only informal supports (e.g. peer support), (3) provides only nonmental health services (e.g. employment and housing), (4) does not provide outpatient services (i.e. only residential or day treatment), and (5) serving only a specific subset of the community (e.g. Indigenous).

#### Agency recruitment

Eleven agencies completed a provincial survey^6^ and met the eligibility criteria for this study. These agencies were invited to participate in this study via email; all responded to the invitation and provided unsolicited reasons for declining. Seven agencies declined to participate; the most common reason for declining was limited capacity (e.g. time and personnel resources). Four agencies agreed to participate. Two of the agencies had data that could not be used. One implemented the MHWCs in 2019 and had a small sample of MHWC clients. The other had separate electronic administrative records for MHWC clients and other agency clients; these records, unfortunately, could not be reliably linked. The remaining two agencies had data that met the guidelines to be used for the study. A formal data-sharing agreement (prepared by The University of Western Ontario research office) was signed by each agency, outlining their consent to participate and data that would be extracted.

#### Data abstraction

Both participating agencies used EMHware (www.emhware.com) as their electronic administration and clinical record system. EMHware personnel did the extraction and de-identification of the data. Agencies varied in when they started using EMHware services and whether, and how, previous data were migrated from other administrative/clinical records systems. As such, the earliest date when electronic service use data were available varied across agencies. The COVID-19 pandemic resulted in a lockdown announcement in Ontario on 17 March 2020, and a halt to all in-person services. Because of this, no data after 17 March 2020, were analyzed.

#### Participating agencies

Agency 1 is located in southwestern Ontario. They implemented their MHWCs in 2013, and electronic data were available since 2008. The MHWCs in Agency 1 are offered from different locations (e.g. agency, community center, and physician office) by child and youth workers, social workers, and registered psychotherapists. Different approaches are used, including narrative therapy, solution-focused therapy, supportive therapy, and other (e.g. emotion-focused therapy, dialectical behavior therapy skills, and motivational interviewing). In this agency, MHWCs were either unscheduled (estimated 85%–90%) or scheduled (estimated 10%–15%) single-session appointments (before COVID-19; personal communication, Agency 1, 2021) and served as a point of intake for agency services (i.e. gathering information, such as presenting problem, to decide what services are offered). Of note, families can complete this intake in other ways (e.g. phone calls). Agency 1 offers a variety of other services along the continuum of care, including workshops, groups, family therapy, individual therapy, and day treatment, among others. The study window for Agency 1 was 6 years and 9 months from June 2013 to March 2020.

Agency 2 is located in southeastern Ontario. They implemented MHWCs in 2006, and electronic data were available since 2016. The MHWCs in Agency 2 are offered at their agency by social workers and registered psychotherapists. Different approaches are used, including narrative therapy, solution-focused therapy, and choice and partnership approach (personal communication, Agency 2, 2021). In this agency, MHWCs were either unscheduled (estimated 85%–90%) or scheduled (estimated 10%–15%) single-session appointments (before COVID-19) and did not serve as a point of intake for agency services. Agency 2 offers a variety of other services along the continuum of care, including groups, family therapy, individual therapy, and in-home intervention. The study window was 3 years and 8 months from June 2016 to March 2020.

#### Inclusion and exclusion criteria for agency clients

There were three inclusion criteria for children and families: (1) had a MHWC visit; (2) children under the age of 16 at the start of the study window, ensuring that they were able to access agency services; and (3) children under the age of 16 at their first MHWC visit. The latter allows families the opportunity to access MHWCs and other agency services even if they did so in the second half of the study window (older youth would have aged-out eligibility for child and youth mental health services).

**Figure 1.**
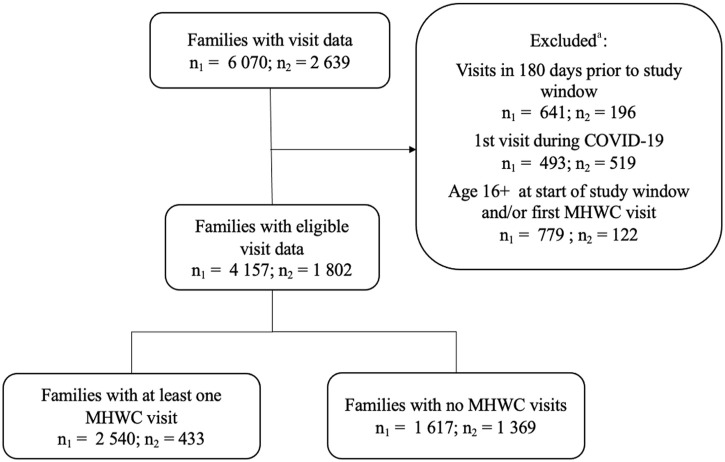
Flow chart showing children/family selection for Agency 1 (n_1_) and Agency 2 (n_2_). MHWC: mental health walk-in clinic. ^a^Criteria applied sequentially in the order shown.

There were three exclusion criteria for children and families: (1) children and families who had visits in the 180 days prior to the study window; thus, all cases included would be starting a new episode of care;^23^ (2) cases with telephone contact only (i.e. no face-to-face or videoconference contacts); and (3) cases where a parent of a child more than 12 years accessed services *without* their child present. For the latter cases, children more than 12 years must consent to have a file opened for them; otherwise, no identifiable information is recorded in the database. Figure 1 presents the sample selection.

### Electronic administrative data

Electronic records (at the participating agencies) consist of every contact that agency staff had with a family or other professional to deliver and coordinate services. This information is entered by agency staff (e.g. clinicians and intake workers) during or shortly after each contact. The information entered for the first MHWC visit was utilized as correlates. Only nonidentifying information was abstracted from the electronic administrative data, including demographics (e.g. child birthdate and gender), visit data (e.g. visit dates and types of contacts), and psychopathology information (e.g. presenting concerns).

#### Correlates

##### Child age

The age, in years, at the time of the first MHWC visit was calculated for each child. Age violated the linearity of the logit assumption (see below) and so was re-coded as a categorical correlate with two categories: <12 years and 12+ years. Children 12+ years were the reference category.

Child gender. The agencies entered child gender using three categories: female, male, and other. The “other” gender category had small cell sizes. As such, it was included in descriptive statistics, but these cases were dropped (n_Agency 1_ = 19; n_Agency 2_ = 2) in the logistic regressions. Female was the reference category.

##### Neighborhood poverty

Neighborhood-level poverty data were utilized given that family income was not available. This variable was derived as follows: (1) the prevalence of low-income households (using the low-income measure) was obtained for each forward sortation area (FSA; i.e. first three digits of the postal code) in Ontario from the 2016 Census;^24^ (2) each FSA was categorized into two-quantiles: high poverty (i.e. LIM = 12.7–47.7; mean = 19.59; SD = 6.11) or low poverty (i.e. LIM = 3.1–12.6; mean = 8.66; SD = 2.52); and (3) the neighborhood poverty two-quantile was assigned to each case based on the family’s postal code.

##### Guardianship

The agencies entered guardianship using seven categories: birth/adoptive father, birth/adoptive mother, birth/adoptive parents, shared custody, grandparents, Children’s Aid Society (i.e. child welfare), and other. Note that shared custody could range from equal custody between parents to predominately one parent. Parents’ relationship status was not available; thus, guardianship with birth/adoptive father or birth/adoptive mother may or may not have reflected a single-parent family. Guardianship at the time of the first MHWC visit was re-coded given small cell sizes: birth/adoptive parents, shared custody, birth/adoptive father or mother, and other. Birth/adoptive parents were the reference category.

##### Presenting concern

The agencies entered presenting concerns from a list of more than 50 options with only minor differences between agencies; coding of multiple concerns was possible. Presenting concern for the first MHWC visit was re-coded into four categories: (a) externalizing concerns, (b) internalizing concerns, (c) parenting and family concerns, and (d) other concerns (e.g. school problems and sleep difficulties). As multiple presenting concerns were often coded, four variables were computed and coded as not a concern or was a concern.

The total number of presenting concern categories was also computed for the first MHWC visit and used as a proxy for complexity and comorbidity. Due to small cell sizes, it was re-coded as a categorical correlate with three categories: 1 presenting concern category, 2 presenting concern categories, and 3+ presenting concern categories, with 3+ presenting concern categories as the reference category.

##### Disposition at discharge

The agencies entered disposition at discharge for the first MHWC visit using six categories. It was re-coded as a categorical correlate with two broad categories: (1) no referral within the agency: no referral was made to services within the agency, *or* referral was made to services outside of the agency (rare; 0.04% of sample); and (2) referral within the agency: anticipate seeing the family again at the agency (see Table S1 in the supplemental materials, for information on the original six categories). Of note, this variable could only be explored with Agency 1.

#### Outcome

Visits were grouped as follows: (1) Non-MHWC visits were grouped into episodes of care. An episode of care was defined as a minimum of three visits with a period of 180 days without visits between episodes.^23^ (2) Children could have visits that did not meet this criterion prior to the first episode of care; these were coded as pre-episode of care visits. (3) Children could also have visits that did not meet this criterion after an episode of care; these are referred to as inter-episode of care visits (i.e. visits between two episodes of care) or (4) post-episode of care visits (i.e. visits after the last episode of care). Figure S1a in the supplemental materials provides a visual representation of how visit data were categorized into the above four groups.

Next, the timing of MHWC use with respect to other agency services was examined. A third of families had more than one MHWC visit (32.3% Agency 1; 36.3% Agency 2; see Table S2 in the supplemental materials). For these families, only the first MHWC visit was considered. This created five possibilities: (1) MHWC use exclusively, (2) MHWC use before other agency services, (3) MHWC use concurrently with other agency services (i.e. *during* a pre-episode of care, episode of care, inter-episode of care, or post-episode of care), (4) MHWC use during the service use trajectory (i.e. *before or after* a pre-episode of care, episode of care, inter-episode of care, or post-episode of care), and (5) MHWC use after other agency services. Figure S1b in the supplemental materials presents examples of these groupings.

The modeling of service use revealed that most families are using MHWCs exclusively or earlier in their service use journey (see “Results”). It also revealed small cell sizes for one or both agencies for MHWC concurrently with other agency services, MHWC during service use trajectory, and MHWC after other agency services. As such, models examining factors that differentiated between all five patterns of MHWC use could not be examined. Instead, two of the five possibilities mentioned above were examined: (1) MHWC use exclusively (reference category) and (2) MHWC use before other agency services.

### Data analyses

Statistical analyses were conducted in SPSS (Version 27) for Windows. All analyses were conducted separately for each agency given differences in how MHWCs were used, when they were implemented, and number of years of data provided.

#### Logistic regression

Logistic regressions were used to identify correlates of MHWC use before other agency services versus MHWC use exclusively. Unadjusted and adjusted odds ratios (ORs) were calculated to determine the effect that each variable had on the outcome independently and adjusting for other variables. ORs can be interpreted as the change in odds of MHWC use before other agency services for every one-unit increase (e.g. 1 year increase in age) or compared with another category (e.g. males compared with females).^25,26^

Correlates were entered in blocks based on the Revised Network-Episode Model categories. The overall model was interpreted first, followed by the individual correlates. Logistic regression was used as the outcome has two levels, and it requires less restrictive assumptions compared with other approaches like discriminant analyses (e.g. homogeneity of variance/covariance).^26^

#### Assumptions

The key assumptions for logistic regression are as follows: linearity of the logit, absence of multicollinearity, and no strongly influencing outliers.^25,26^ First, linearity of the logit specifies that there should be a linear relationship between continuous correlates and their logit-transformed outcomes.^25,26^ The Box-Tidwell test was conducted to examine this assumption. Child age violated this assumption, and so the variable was dichotomized. Second, absence of multicollinearity among independent variables was examined by computing correlations between the correlates and no issues emerged.^25,26^ Finally, strongly influential outliers were not an issue in this study as all correlates were categorical.^25,26^

#### Missing data

Multiple imputation (40 imputations) was used to handle missing data for the correlates used in the logistic regressions. Multiple imputation was conducted separately for each agency. Overall, 66.7% and 59.2% of families in Agency 1 and Agency 2, respectively, had no missing data across correlates (see Table S3 in the supplemental materials, for more information).

## Results

Table 1 presents the descriptive statistics for all families with at least one MHWC visit prior to multiple imputation. Between 36.8% and 39.0% of the sample was age 12 and over, and between 42.3% and 50.0% were females. There were statistically significant differences between the two agencies in all variables, except for child’s age.

**Table 1.** Descriptive statistics of children, family, and service use for all families with a MHWC visit.

	Agency 1 (*N* = 2540)	Agency 2 (*N* = 433)
	*n* (%)	*n* (%)
Child		
Child age		
<12 years	1606 (63.2)	264 (61.0)
12+ years	934 (36.8)	169 (39.0)
Child gender		
Female	1271 (50.0)*	183 (42.3)*
Male	1248 (49.1)	223 (51.5)
Other	19 (0.7)	2 (0.5)
Missing	2 (0.1)*	25 (5.8)*
Family		
Guardianship of child		
Birth/adoptive parents	812 (32.0)	150 (36.7)
Birth/adoptive mother	591 (23.3)	113 (26.1)
Birth/adoptive father	101 (4.0)	20 (4.6)
Shared custody	425 (16.7)*	45 (10.4)*
Grandparents	85 (3.3)	20 (4.6)
CAS^a^	30 (1.2)	6 (1.4)
Other	25 (1.0)	6 (1.4)
Missing	471 (18.5)	64 (14.8)
Neighborhood poverty		
Low poverty	1789 (70.4)*	259 (59.8)*
High poverty	733 (28.9)*	161 (37.2)*
Missing	18 (0.7)*	13 (3.0)*
Service use		
Presenting concern^b^		
Externalizing	806 (31.7)	171 (39.5)
Internalizing	1292 (50.9)*	236 (54.5)*
Parenting and family	639 (25.2)*	57 (13.2)*
Other	500 (19.7)*	236 (54.5)*
Missing	717 (28.2)*	51 (11.8)*
Number of presenting concerns		
1	786 (30.9)	161 (37.2)
2	725 (28.5)	136 (31.4)
3	247 (9.7)*	73 (16.9)*
4	65 (2.6)	12 (2.8)
Missing	717 (28.2)*	51 (11.8)*
Disposition at discharge		
No referral within the agency	1517 (59.7)	N/A
Referral within the agency	961 (37.8)	N/A
Missing	62 (2.4)	N/A

MHWC: mental health walk-in clinics; CAS: Children’s Aid Society.

Descriptive statistics between the two agencies were compared using chi-square tests and *z-*pairwise tests (if chi-square tests were significant to determine which proportion was different).^a^In Ontario, CAS provide child welfare services.

b Clinicians can code multiple presenting concerns for a visit.

* *p* < .05

### Use of MHWCs and other agency services

For Agency 1, 33.2% of families used only MHWCs and no other agency services. Of these families, 81.4% had only one MHWC visit, and the remaining had two or more MHWC visits (see Table S2 in the supplemental materials). The remainder, 66.8%, of families accessed both MHWCs and other agency services. These families accessed the MHWCs at different time points (see Table 1 and Figure S1b). The majority of cases, 56.3%, had their first MHWC visit before other agency services, 1.6% concurrently with other agency services, 5.7% during their service use trajectory (but not concurrently with other services), and 3.2% after other agency services. As the later three patterns had small cell sizes, these could not be included in the regression model.

For Agency 2, 43.0% of families used only MHWCs and no other agency services. Of these families, 76.9% had only one MHWC visit, and the remaining had two or more MHWC visits (see Table S2 in the supplemental materials). The remainder, 57.0%, of families accessed both MHWCs and other agency services. More specifically, 25.4% had their first MHWC visit before other agency services, 20.8% concurrently with other agency services, 4.2% during their service use trajectory (but not concurrently with other services), and 6.7% after other agency services. As the later three patterns had small cell sizes, these could not be included in the regression model.

### Variables correlated with MHWC use before other agency services

For Agency 1, the full binary regression model predicting MHWC use before other agency services (vs MHWC use exclusively) provided an adequate fit based on the omnibus test (*p* < .01). The social content block and illness career block each independently provided an adequate fit. In the multivariate model, children <12 years had 25% lower odds of MHWC use before other agency services, compared with children 12+ years. Children whose disposition at discharge was “no referral within the agency” had 95% lower odds of MHWC use before other agency services, compared with children whose disposition at discharge was “referral within the agency” (see Table 2). This model was re-run without disposition at discharge to be better able to compare the findings to Agency 2 (see Table S4 in the supplemental materials). There were no substantial differences in the results.

**Table 2.** Unadjusted and adjusted odds ratios for correlates of MHWC use before other agency services versus MHWC use only.

Social content	Agency 1		Agency 2	
	Unadjusted OR (95% CI)	Adjusted OR (95% CI)	Unadjusted OR (95% CI)	Adjusted OR (95% CI)
Child age^a^				
<12 years	0.70 (0.59–0.84)**	0.75 (0.61–0.93)*	1.27 (0.77–2.08)	1.32 (0.77–2.29)
Child gender^b^				
Male	0.89 (0.75–1.06)	1.03 (0.80–1.32)	0.76 (0.46–1.25)	0.67 (0.38–1.16)
Neighborhood poverty^c^				
High poverty	1.21 (0.99–1.46)	1.15 (0.87–1.52)	1.22 (0.74–2.02)	1.13 (0.66–1.89)
Guardianship of child^d^				
Shared custody	0.93 (0.71–1.21)	1.02 (0.70–1.48)	2.73 (1.18–6.27)*	2.87 (1.19–6.95)*
Birth/adoptive mother or father	1.13 (0.90–1.43)	1.20 (0.87–1.67)	1.57 (0.88–2.80)	1.56 (0.84–2.91)
Other	1.47 (0.95–2.26)	1.78 (0.98–3.21)	1.40 (0.43–4.60)	1.48 (0.43–5.12)
Presenting concern				
Externalizing^e^	1.25 (0.93–1.68)	1.21 (0.59–2.49)	1.06 (0.64–1.76)	1.06 (0.40–2.85)
Internalizing^e^	1.34 (1.05–1.70)*	1.00 (0.48–2.10)	0.67 (0.41–1.11)	0.65 (0.25–1.68)
Parenting and family^e^	0.78 (0.61–0.99)*	0.79 (0.37–1.67)	1.11 (0.59–2.11)	1.04 (0.40–2.70)
Other^e^	1.17 (0.83–1.67)	1.21 (0.59–2.47)	0.83 (0.50–1.38)	0.77 (0.30–2.02)
Number of presenting concerns^f^				
1	0.74 (0.48–1.15)	0.77 (0.22–2.67)	1.26 (0.65–2.44)	1.02 (0.17–6.05)
2	0.85 (0.54–1.34)	0.80 (0.36–1.76)	0.80 (0.39–1.63)	0.68 (0.22–2.18)
Disposition at discharge^g^				
No referral within the agency	0.05 (0.04–0.07)**	0.05 (0.03–0.07)**	—	—

MHWC: mental health walk-in clinics; OR: odds ratio; CI: confidence interval.

a Reference category is children 12+ years.

b Reference category is females.

c Reference category is low poverty.

d Reference category is birth/adoptive parents.

e Reference category is no presenting problem in that category.

f Reference category 3+ presenting concern categories.

g Reference category is referral within the agency.

* *p* < .05; **p < .01.

For Agency 2, the full binary regression model predicting MHWC use before other agency services (vs MHWC use exclusively) did not provided an adequate fit based on the omnibus test. Of note, only the social content block could be tested due to data availability. In the multivariate model, children whose parents have shared custody had 187% higher odds of MHWC use before other agency services, compared with living with two birth/adoptive parents (see Table 2).

Descriptive statistics for the subsample used in the regressions following multiple imputation procedures are presented in Table S5 in the supplemental materials.

## Supplementary analyses

Age, guardianship, and disposition at discharge were significant correlates of time to a second visit. It is possible that there are differences with respect to presenting concerns within these subgroups (e.g. younger children have more externalizing problems, whereas older children have more internalizing problems). As such, these differences were explored.

For Agency 1, children 12+ years had significantly more internalizing problems, while children <12 years had more externalizing problems. Moreover, children whose disposition at discharge was “no referral within the agency” had more parenting and family problems, while children whose disposition at discharge was “referral within the agency” had more internalizing problems (see Tables 3 and 4).

**Table 3.** Relationship between presenting concerns and child age for Agency 1.

	Child age	
	<12 years (%)	12+ years (%)
Externalizing	50.2	35.3*
Internalizing	58.7	69.9*
Parenting and family	40.1	36.4
Other	30.0	34.6

The table summarizes the percentages of families with a presenting concern category within an age group. Column percentages are reported; as families could have had more than one presenting concern, percentages do not sum to 100. For example, 50.2% of children <12 years presented with externalizing problems, whereas 35.3% of children 12+ years presented with externalizing problems. The difference in externalizing problems between <12-year-old and 12+ year-old groups (i.e. 50.2% vs 35.3%) is statistically significant which is denoted by the asterisk.

* *p* < .01.

**Table 4.** Relationship between presenting concerns and disposition at discharge for Agency 1.

	Disposition at discharge	
	No referral within the agency (%)	Referral within the agency (%)
Externalizing	43.6	45.9
Internalizing	57.0	73.3*
Parenting and family	42.5	32.2*
Other	33.5	28.8

The table summarizes the percentages of families with a presenting concern category within a disposition at discharge group. Column percentages are reported; as families could have had more than one presenting concern, percentages do not sum to 100. For example, 57.0% of children with “no referral within the agency” presented with internalizing problems, whereas 73.3% of children with “referral within the agency” presented with internalizing problems. The difference in internalizing problems between “no referral within the agency” and “referral within the agency” groups (i.e. 57.0% vs 73.3%) is statistically significant, which is denoted by the asterisk.

* *p* < .01.

For Agency 2, the percentage presenting with parenting and family problems was higher in children with shared custody arrangements compared with those with birth/adoptive parents, but this difference was not statistically significant (see Table 5).

**Table 5.** Relationship between presenting concerns and guardianship for Agency 2.

	Guardianship	
	Birth/adoptive parents (%)	Shared custody (%)
Externalizing	39.7	34.0
Internalizing	58.5	50.0
Parenting and family	18.1	30.4
Other	58.8	71.4

The table summarizes the percentages of families with a presenting concern category within a guardianship group. Column percentages are reported; as families could have had more than one presenting concern, percentages do not sum to 100. For example, 39.7% of children under the guardianship of both parents presented with externalizing problems, whereas 34.0% of children in shared custody presented with externalizing problems. The difference in externalizing problems between children under the guardianship of both parents and children in shared custody groups (i.e. 39.7% vs 34.0%) is not statistically significant.

## Discussion

This study explored how MHWC use is related to use of other agency services. A substantial number of families use MHWCs alongside other services (57%–67%). As hypothesized, families tended to use MHWCs earlier in their service use journey with some differences between agencies. In Agency 1, more families used MHWCs before other agency services (67% vs 57%), while in Agency 2, more families used MHWCs concurrently with other agency services (21% vs 1.6%). One of the differences between the agencies that may account for this is the use of MHWCs as a point of intake in Agency 1, but not in Agency 2.

Correlates of MHWC use before other agency services differed between the agencies. For Agency 1, younger children had lower odds of MHWC use before other agency services. This is consistent with some studies examining service use.^27^ Supplementary analyses showed that younger children (<12 years) had more externalizing problems whereas older children (12+ years) had more internalizing problems. The data suggest that it is likely that agencies are using more behavioral approaches with younger children and children with externalizing problems (which tend to co-occur), and more cognitive approaches with older children and children with internalizing problems (which also tend to co-occur). Behavioral approaches (e.g. praise, rewards, and behavioral activation) may be easier to cover in a MHWC setting and for families to implement following a MHWC session. So, these families may be less likely to need other agency services. It is also possible that these behavioral approaches require parents to change *their* behavior, which they may not be prepared to do (Informed by the discussion with Agency 1 and Agency 2 when the findings were presented to them in June 2022). So, these families may be less likely to seek other agency services. Cognitive approaches (e.g. cognitive restructuring), on the contrary, may require more ongoing support. It also tends to require more child/youth change, which may be easier for parents to support and/or for the youth to seek independently (Informed by the discussion with Agency 1 and Agency 2 when the findings were presented to them in June 2022). Thus, it may be an interaction of age, presenting concern, and therapeutic approach that impacts and influences service use.

Children whose disposition at discharge was “no referral within the agency” had lower odds of using other agency services after MHWCs, compared with children whose disposition at discharge was “referral within the agency.” Supplementary analyses showed that children whose disposition at discharge was “no referral within the agency” had more parenting and family and other problems, while children whose disposition at discharge was “referral within the agency” had more internalizing problems. It may be that internalizing problems represent more severe psychopathology, thereby, needing additional supports. However, information about the severity of the presenting concern was not recorded so as to examine this possibility.

For Agency 2, children whose parents have shared custody had higher odds of MHWC use before other agency services compared with those living with birth/adoptive parents. Supplementary analyses did not elucidate any statistically significant relationship between guardianship and presenting problems, which may have been due to the smaller sample size in Agency 2. Children or families in these living arrangements may be exposed to, or at greater risk of, more stressors (e.g. parental conflict during separation), which is consistent with the trend of more parenting and family issues as presenting problems. As such, these families may require more supports.^28,29^

### Implications and limitations

Between 33% and 43% of families use MHWCs exclusively. Thus, it appears that MHWCs were sufficient for these families, and potentially easing the pressure on other agency services. The remaining families (57%–67%) use MHWCs alongside other agency services. For these families, MHWC use was most often earlier in the service use trajectory, that is, before or concurrently with other agency services. Thus, MHWCs most often serve as the first service with an agency and as support while they are connected to other agency services. MHWCs were also used after other agency services, where they may serve as booster sessions (e.g. reviewing previously learned skills to maintain treatment gains); however, this was rare (3%–7%).

There were some limitations that are worth noting. First, there was a substantial list of inclusion and exclusion criteria. As such, the results are only generalizable to agencies similar to those that were recruited. One of the criteria required that agencies be located in a census division with a small urban center. The use of MHWCs may be different in agencies located in other census divisions. It is possible that, in larger urban centers, families have a MHWC visit at one agency and then other visits at another agency. This would result in different patterns of service use within a given agency. Another of the criteria required that agencies serve most mental health problems. The use of MHWCs may vary in agencies that specialize in some presenting concerns, like addictions. It is difficult to say, however, exactly how this and other criteria (e.g. age) would impact the results. Second, the number of families that used MHWCs concurrently, during the service use trajectory, or after other agency services was relatively small. As such, correlates specific to these time points could not be explored. Third, logistic regression, unlike other statistical approaches (e.g. Cox regression), does not take censoring into account.^25,26,30^ Fourth, information about the severity and/or impairment of the presenting concern was not available. This information would be helpful in further understanding the findings. Fifth, children more than 12 years must consent to have a file opened for them. This means that files are not opened (i.e. no information is recorded) when a parent of a child more than 12 years accesses a MHWC *without* their child present. As such, these families could not be included or accounted for. Sixth, this study focused on two community mental health agencies. Other services from the educational system, justice system, health care system, and private practice were not captured in this study. Thus, it is possible that some children/families accessed services from multiple sources, which could decrease the need for additional service at the CYMH agency. However, it is also important to note that only one case (0.04%) was referred to an external agency for services. The extremely low rate of referral to external agencies confirms the rationale for our choice of agencies; of families that came to the MHWCs, virtually all received help within the agency. This information was only available for Agency 1, and the referral rate may have been different for Agency 2. Finally, it is unclear how the differences between the agencies (e.g. length of study window, whether MHWCs are used as a point of intake) lead to differences in correlates.

## Conclusion

MHWCs are a model of service delivery that has gained increasing interest and traction. Studies on MHWCs are promising, showing perceived improvement immediately after the MHWC session and at follow-up. There is limited research examining how MHWCs relates to other services families may access. This study begins to fill this gap in the literature. Overall, more than half of families used MHWCs and other agency services before or concurrently with other agency services. Child age, guardianship, and disposition at discharge emerged as correlates of MHWC use before other agency services.

## Supplemental Material

sj-docx-1-map-10.1177_27550834231186682 – Supplemental material for Accessing mental health walk-in clinics and other services for children and families Click here for additional data file. Supplemental material, sj-docx-1-map-10.1177_27550834231186682 for Accessing mental health walk-in clinics and other services for children and families by Catalina Sarmiento and Graham J Reid in The Journal of Medicine Access

## Declarations

**Ethics approval and consent to participate:** The study received ethics approval by The University of Western Ontario Research Ethics Board (#115545). The participating agencies signed agreements specifying the data that would be provided for the project and dissemination of findings.

**Consent for publication:** Not applicable.

**Availability of data and materials:** The participating agencies did not consent for their data to be shared publicly, so supporting data are not available.
