# Supplementary material for: Accessing mental health walk-in clinics and other services for children and families
Source: J Med Access. 2023 Jul 31;7:27550834231186682. doi: 10.1177/27550834231186682 (PMC10392159; doi:10.1177/27550834231186682)
Supplement: sj-docx-1-map-10.1177_27550834231186682 – Supplemental material for Accessing mental health walk-in clinics and other services for children and families [file sj-docx-1-map-10.1177_27550834231186682.docx]

Supplemental Materials

**Table S1.** Disposition at discharge subcategories after first MHWC visit for all families with a MHWC visit.

|  | Agency 1  (N=2540) |
| --- | --- |
|  | n (%) |
| No referral within the agency | 1517 (59.7%) |
| Mutual completion | 745 (29.3%) |
| No referral | 771 (30.4%) |
| Referral to external agency | 1 (0.04%) |
| Referral within the agency | 961 (37.8%) |
| Still waiting for another service | 5 (0.2%) |
| Referral within the agency | 945 (37.2%) |
| Wanting additional services and directed to intake | 11 (0.4%) |
| Missing | 62 (2.4%) |

**Table S2.** Number of MHWC visits for families with MHWC and other agency service use, and MHWC use exclusively.

|  | Number of MHWC visits  n (%) | | | | | | | | | |
| --- | --- | --- | --- | --- | --- | --- | --- | --- | --- | --- |
|  | 1 | 2 | 3 | 4 | 5 | 6 | 7 | 8 | 9 | 10 |
| Agency 1 | |  |  |  |  |  |  |  |  |  |
| MHWC and other agency service use | 1033 | 413 | 168 | 46 | 21 | 8 | 3 | 1 | 2 | 1 |
|  | (60.9%) | (24.4%) | (9.9%) | (2.7%) | (1.2%) | (0.5%) | (0.2%) | (0.1%) | (0.1%) | (0.1%) |
| MHWC use exclusively | 687 | 127 | 20 | 7 | 2 | 1 | 0 | 0 | 0 | 0 |
|  | (81.4%) | (15.0%) | (2.4%) | (0.8%) | (0.2%) | (0.1%) | (0.0%) | (0.0%) | (0.0%) | (0.0%) |
| Agency 2 | |  |  |  |  |  |  |  |  |  |
| MHWC and other agency service use | 133 | 61 | 23 | 13 | 6 | 4 | 4 | 3 | 0 | 0 |
|  | (53.8%) | (24.7%) | (9.3%) | (5.3%) | (2.4%) | (1.6%) | (1.6%) | (1.2%) | (0.0%) | (0.0%) |
| MHWC use exclusively | 143 | 31 | 4 | 3 | 5 | 0 | 0 | 0 | 0 | 0 |
|  | (76.9%) | (16.7%) | (2.2%) | (1.6%) | (2.7%) | (0.0%) | (0.0%) | (0.0%) | (0.0%) | (0.0%) |

**Table S3.** Analyses of missing data for the logistic regression models predicting MHWC use before other agency services, versus MHWC use only.

| Total missing across correlates | Agency 1 | Agency 2 |
| --- | --- | --- |
|  | n (%) | n (%) |
| No missing data | 1504 (66.7%) | 174 (59.2%) |
| 1 | 313 (13.9%) | 97 (33.0%) |
| 2 | 415 (18.4%) | 10 (3.4%) |
| 3 | 23 (1.0%) | 3 (1.0%) |
| 4 | 1 (0.04%) | 2 (0.7%) |
| 5 | - | 8 (2.7%) |

*Note.* Analyses by families/cases (e.g., 13.9% of families were missing data on 1 correlate for Agency 1).

**Table S4.** Unadjusted and adjusted odds ratios for correlates of MHWC use before other agency services versus MHWC use only without disposition at discharge.

|  | Agency 1 | |  | Agency 2 | |
| --- | --- | --- | --- | --- | --- |
|  | Unadjusted  OR (95% CI) | Adjusted  OR (95% CI) |  | Unadjusted  OR (95% CI) | Adjusted  OR (95% CI) |
| Social content |  |  |  |  |  |
| Child age ^a^ |  |  |  |  |  |
| < 12 years old | 0.70  (0.59-0.84)** | 0.70  (0.58-0.85)** |  | 1.26  (0.76-2.09) | 1.37  (0.78-2.38) |
| Child gender ^b^ |  |  |  |  |  |
| Male | 0.89  (0.75-1.06) | 0.93  (0.77-1.13) |  | 0.74  (0.45-1.21) | 0.62  (0.36-1.09) |
| Neighbourhood poverty ^c^ |  |  |  |  |  |
| High poverty | 1.21  (0.99-1.46) | 1.15  (0.92-1.44) |  | 1.25  (0.75-2.07) | 1.13  (0.66-1.94) |
| Guardianship of child ^d^ |  |  |  |  |  |
| Shared custody | 0.93  (0.71-1.21) | 1.02  (0.75-1.43) |  | 3.03  (1.25-7.35)* | 3.26  (1.33-8.13)* |
| Birth/adoptive mother or father | 1.13  (0.90-1.43) | 1.15  (0.88-1.51) |  | 1.53  (0.84-2.77) | 1.55  (0.83-2.91) |
| Other | 1.47  (0.95-2.26) | 1.50  (0.92-2.43) |  | 1.31  (0.42-4.10) | 1.41  (0.42-4.72) |
| Presenting concern |  |  |  |  |  |
| Externalizing ^e^ | 1.25  (0.93-1.68) | 1.50  (0.95-2.37) |  | 1.23  (0.68-1.87) | 1.12  (0.39-3.22) |
| Internalizing ^e^ | 1.34  (1.05-1.70)* | 1.40  (0.86-2.26) |  | 0.68  (0.41-1.31) | 0.64  0.22-1.85) |
| Parenting and family ^e^ | 0.78  (0.61-0.99)* | 0.78  (0.50-1.20) |  | 1.18  (0.62-2.23) | 1.04  (0.36-3.06) |
| Other ^e^ | 1.17  (0.83-1.67) | 1.14  (0.74-1.74) |  | 0.91  (0.54-1.52) | 0.84  (0.29-2.43) |
| Number of presenting concerns ^f^ |  |  |  |  |  |
| 1 | 0.74  (0.48-1.15) | 0.91  (0.40-2.08) |  | 1.11  (0.57-2.18) | 0.96  (0.13-7.17) |
| 2 | 0.85  (0.54-1.34) | 0.93  (0.54-1.61) |  | 0.71  (0.34-1.47) | 0.62  (0.17-2.20) |

* *p* < .05 ** *p* < .01

^a^ Reference category is children 12+.

^b^ Reference category is females.

^c^ Reference category is low poverty.

^d^ Reference category is birth/adoptive parents.

^e^ Reference category is no presenting problem in that category.

^f^ Reference category 3+ presenting concern categories.

**Table S5.** Descriptive statistics of child, family, and service use for families included in the logistic regression, following multiple imputation.

|  | Agency 1 | Agency 2 |
| --- | --- | --- |
|  | n (%) | n (%) |
| ***Child*** |  |  |
| Child age |  |  |
| <12 years old | 1385 (61.4%) | 176 (61.5%) |
| 12+ years old | 871 (38.6%) | 110 (38.5%) |
| Child gender |  |  |
| Female | 1166.3 (51.7%) | 115.4 (40.3%) |
| Male | 1089.7 (48.3%) | 170.6 (59.7%) |
| ***Family*** |  |  |
| Guardianship of child |  |  |
| Birth/adoptive parents | 924.9 (41.0%) | 132.5 (46.2%) |
| Shared custody | 431.9 (19.1%) | 38.4 (13.4%) |
| Birth/adoptive mother/ father | 744.3 (33.0%) | 98.2 (34.3%) |
| Other | 155 (6.9%) | 17 (5.9%) |
| Neighborhood poverty |  |  |
| Low poverty | 1614 (71.5%) | 181.8 (63.6%) |
| High poverty | 642 (28.5%) | 104.2 (36.4%) |
| ***Service use*** |  |  |
| Presenting concern^1^ |  |  |
| Externalizing | 1002.1 (44.4%) | 118.1 (41.3%) |
| Internalizing | 1421 (63.0%) | 162.8 (56.9%) |
| Parenting and family | 873.3 (38.7%) | 56.5 (19.8%) |
| Other | 717.6 (31.8%) | 168.5 (58.9%) |
| Number of presenting concerns |  |  |
| 1 | 1016.4 (45.1%) | 134 (46.9%) |
| 2 | 792.9 (35.1%) | 88.7 (31.0%) |
| 3+ | 446.8 (19.8%) | 63.3 (22.1%) |
| Disposition at discharge |  |  |
| No referral within the agency | 1429.5 (63.4%) | N/A |
| Referral within the agency | 826.5 (36.6%) | N/A |

^1^ Clinicians could code multiple presenting concerns for a visit.

**Figure S1a.** Visual representation of how visit data were categorized with respect to episodes of care. An episode of care was defined as a minimum of 3 visits with a period of 180 days without visits between episodes (see child/family 4). Children could have visits that did not meet this criterion prior to the first episode of care; these were coded as pre-episode of care visits (see child/family 3). Children could also have visits that did not meet this criterion after an episode of care; these are referred to as inter-episode of care visits (i.e., visits between two episodes of care; see child/family 2) or post-episode of care visits (i.e., visits after the last episode of care; see child/family 1).

**Figure S1b.** Visual representation of the timing of the first MHWC visit with respect to other agency services. This created five possibilities: (1) MHWC use exclusively, (2) MHWC use before other agency services, (3) MHWC use during the service use trajectory (i.e., *before or after* a pre-episode of care, episode of care, inter-episode of care, or post-episode of care), (4) MHWC use concurrently with other agency services (i.e., *during* a pre-episode of care, episode of care, inter-episode of care, or post-episode of care), and (5) MHWC use after other agency services.
